# Optical Power Scale Realization by Laser Calorimeter after 45 Years of Operation

**DOI:** 10.6028/jres.126.011

**Published:** 2021-06-28

**Authors:** Matthew T. Spidell, Anna K. Vaskuri

**Affiliations:** 1 National Institute of Standards and Technology, Boulder, CO 80305, USA

**Keywords:** calorimetry, laser power, primary standard

## Abstract

To calibrate laser power and energy meters, the National Institute of Standards and Technology (NIST) uses several detector-based realizations of the scale for optical radiant flux; these realizations are appropriate for specific laser power/energy ranges and optical coupling configurations. Calibrations from 1 µW to 2 W are currently based upon calorimeters. Validation by comparisons against other primary representations of the optical watt over the last two decades suggests the instruments operate well within their typical reported uncertainty level of 0.86 % with 95 % confidence. The dominant uncertainty contribution in the instrument is attributable to light scattered by the legacy window, which was not previously recognized. The inherent electro-optical inequivalence in the calorimeter’s response was reassessed by thermal modeling to be 0.03 %. The principal contributions to the overall inequivalence were corrected, yielding a shift in scale representation under 0.2 % for typical calibrations. With updates in several uncertainty contributions resulting from this reassessment, the resulting combined expanded uncertainty (k = 2) is 0.84 %, which is essentially unchanged from the previous result provided to calibration customers.

## 1 Introduction

Constructed between 1973 and 1975, the C4 isoperibol calorimeters [1] have been in continuous use at the National Institute of Standards and Technology (NIST) to provide laser power meter calibration services to commercial, institutional, and Department of Defense users through customer-owned transfer standards. These instruments directly represent 100 µW to 200 mW over a wavelength range of 325 nm to 2 µm and serve as the basis for range expansion techniques, typically using in-situ calibrated optical beamsplitters and attenuators, to achieve a calibration power range of 1 µW to 2 W.

As originally constructed, the C4 instruments provided ~1% uncertainty at a 95% confidence interval during typical calibrations [2], which generally met user requirements. One notable exception is the Laser Interferometer Gravitational Wave Observatory photon calibrator program, which anticipates a need for scale realization uncertainty an order of magnitude lower than that currently achieved by the measurement system [3]. The scale realization provided by the C4 instruments has been validated using radiation pressure and cryogenic radiometry, with disagreements equal to less than half of the expanded uncertainty level [4].

## 2 Detector-Based Representation of the Optical Watt

**Fig. 1:**
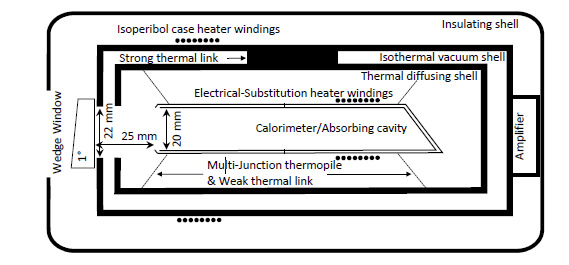
A cross section of the C4 instrument.

The C4 instruments are based upon a cavity calorimeter that is isolated in a temperature-controlled vacuum enclosure, as shown in Fig. 1. The cavity geometry is optimized for low electro-optical inequivalence and retains its calibration factor over time [5]. These electrically calibrated standards are traceable to the International System of Units (SI) through the volt, ohm, and second transfer standards to achieve the joule and watt [6, 7]. The calorimeter is electrically calibrated by injecting a known amount of energy into the resistive heater windings and equating that energy to a corrected thermal rise.

**Fig. 2:**
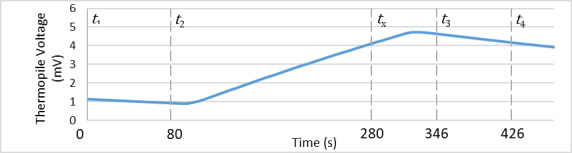
Thermopile voltage for the C4 calorimeter during a typical measurement sequence with approximately 110 mW injected for 200 s starting at *t* = 80 s.

A cavity absorber suspended in a heated vacuum enclosure constitutes the calorimeter and is shown in Fig. 1. As described in Ref. [8], the calorimeter may be modeled as a two-time-constant system. The first time constant, τ, is associated with the cooling rate of the calorimeter (with respect to its vacuum enclosure). The second time constant, $\tau_{1}$, is associated with the rate at which heat flows across the calorimeter mass and is roughly an order of magnitude smaller than the first time constant for the geometry shown in Fig. 1. During a typical measurement sequence (illustrated in Fig. 2), the calorimeter is first preheated, and then the temperature distribution across the calorimeter is allowed to propagate for a multiple of at least seven $\tau_{1}$ [8] before the initial rating period ($t_{1}\leq t\leq t_{2}$). During the injection period ($t_{2}\leq t\leq t_{x}$), the calorimeter may be heated either optically to measure laser power or electrically to establish the calorimeter’s calibration constant. Following a postinjection wait period of at least seven $\tau_{1}$, there is a final rating period ($t_{3}\leq t\leq t_{4}$).

The corrected temperature rise is assessed using a one-time-constant model [8]. The calorimeter case is held at a constant temperature of 35 °C using a resistance bridge, as described in Ref. [5]. The steady-state thermopile voltage, $V_{\infty }$, is nonzero because of radiative heat flow through the vacuum window. The term $V_{\infty }$ is obtained by fitting the one-time-constant model to the initial ($t_{1}\leq t\leq t_{2}$) and final ($t_{3}\leq t\leq t_{4}$) rating periods of the calorimeter’s time-dependent response:

**Table:** 

Vt=V1∙exp-G∙t-t1+V∞ , for t1≤t≤t2V4∙exp-G∙t-t4+V∞ , for t3≤t≤t4	(1)

where $V_{1}$ and $V_{4}$ are initial voltages, and G=1/τ is the first inverse time constant.

The corrected temperature rise, $\Delta V_{T}$, expressed in volts, is defined as

**Table:** 

$\Delta V_{T}=V\left(t\right)-V\left(t_{2}\right)+\int_{t_{2}}^{t}V\left(\overset{-}{t}\right)\;d\overset{-}{t}\;,\;\text{for}\;t>t_{3}$	(2)

When the one-time-constant model is valid, $\Delta V_{T}$ is constant. This may be used to validate the multiple of $\tau_{1}$ allowed for heat to propagate within the cavity.

## 3 Validation of Scale Representation

**Fig. 3:**
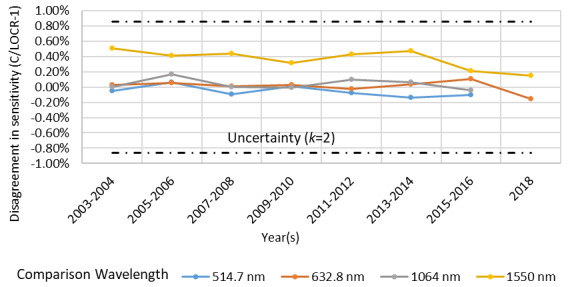
Disagreement in sensitivity of the C4 instruments compared to NIST’s LOCR.

The C4 instrument is compared annually to a laser optimized cryogenic radiometer (LOCR) [9, 10], with the comparison results shown in Fig. 3. The comparison results are then used to validate the electrical and optical characteristics of the calorimeter as well as the measurement software. The results of these comparisons did not suggest a systematic electro-optical inequivalence to previous operators. At a wavelength of 1550 nm, measurements prior to 2015 in the C4 calorimeter laboratory and LOCR laboratory utilized sources with differing linewidths. Because the photodiode transfer standards used to compare values from these laboratories have spectrally dependent sensitivity, the differing linewidths yielded inequivalence of approximately 0.3% to 0.5%. The results of measurements after 2015, employing the same source, reduced the apparent discrepancy to less than 0.2%.

## 4 Window Occlusion by Vacuum Grease

In May of 2019, a dispersion of grease on the inner surface of the vacuum window of the C4-1 instrument was noticed during routine use. When inspected, the C4-4 instrument was found to suffer from the same deficiency. There was no step change in the calorimeter responsivity from 2003 to 2019 to herald this occlusion. As such, the grease has likely been in the optical path since the first comparisons of the C-calorimeter against cryogenic radiometer standards in 2003. We do not know when the grease entered the optical path. With the instrument’s case held at 35 °C to meet isoperibol conditions for the calorimeter [11], we assume more volatile components of the grease condensed on the moderately cooler window. While we did not quantify the effect on transmission prior to cleaning, a fine dispersion of vacuum oil was separately demonstrated to affect transmissivity at the 1% level [12].

**Fig. 4:**
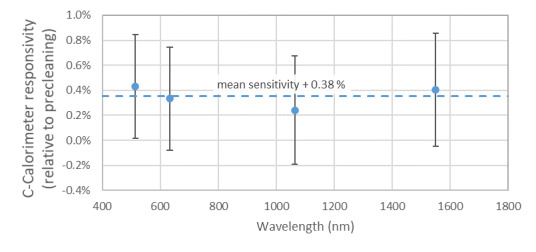
Relative responsivity of the calorimeter after cleaning when compared to precleaning responsivity. Error bars are at *k* = 2.

The vacuum grease was removed by strong solvents and additional cleaning following standard techniques to achieve a contaminant-free surface under visual inspection. The window was returned to the calorimeter. Reassembly without vacuum grease has not caused a deficiency in the vacuum level achieved (typically 10^−7^ torr or lower). With the window clean, we re-evaluated the calorimeter sensitivity using the typical ensemble of transfer standards [10]. Results demonstrated that the relative responsivity of the calorimeter increased by approximately 0.4%, as shown in Fig. 4, suggesting a potential error in one or more of the terms used to describe the calorimeter (window transmissivity, cavity absorptivity, and corrected temperature rise calibration), prompting a re-evaluation.

## 5 Electrical and Optical Characteristics

Laser power reported by the C4 instruments is postprocessed from the raw signal [13] using an analytical package with the electrical and optical characteristics of the calorimeter. These characteristics are: (1) the relationship between corrected temperature rise [11] and injected electrical energy, (2) window spectral transmissivity, (3) cavity absorptivity, and (4) electro-optical inequivalence. The relationship between corrected temperature rise and injected electrical energy has been stable over the previous two decades and is detailed in Refs. [11] and [13]. The remaining terms are examined in detail here.

### 5.1 Cavity Absorptivity Term

The C4 calorimeter interior surface is a 90 mm long, 20 mm diameter cylinder with its rear surface angled at 30°. This geometry causes the principal ray to undergo multiple internal reflections off of a diffuse black paint, achieving high absorptivity [1, 5]. The calorimeter is employed with the laser centered and normal to the cavity, underfilling the aperture. Light rejected by the cavity is emitted into the full hemisphere. The absorptivity coefficient of the calorimeter cavity was previously established at 0.9998 analytically [1]. This term was previously validated by measuring the backscatter of known incident power at 676 nm [5, 14]. While the method is generally effective, and the errors can be largely controlled analytically, the assumptions with respect to thermal partitioning yield increased uncertainty. No description of the spectral absorptivity at other wavelengths was provided in previous work [14]. The legacy analytical software package used by the C4 instruments employed an absorptivity value of 0.9962. A thorough examination of the instrument’s analytical package failed to identify any documentation supporting this value.

**Fig. 5:**
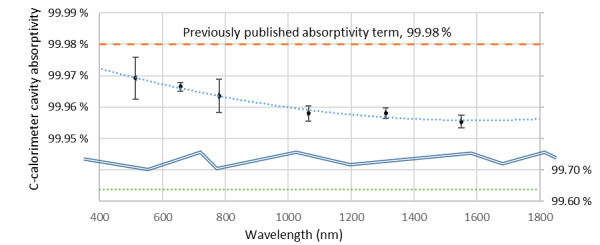
Calorimeter absorptivity. Error bars show *k* = 2 uncertainty level for absorptivity measurements.

Directional-hemispherical reflectance measurements of an *identical* cavity (removed from its vacuum chamber) using a Taylor-type absolute reflectance scheme are appropriate to measure reflectance for this illumination condition [15]. An implementation of this method, described in Ref. [16], was utilized for this measurement series at several wavelengths currently preferred by calibration users. The measurement results are plotted in Fig. 5 along with the previous value used in the legacy analytical software package as well as the previously published value. The modest spectral dependence is most likely attributable to the spectral absorptivity of the paint.

### 5.2 Window Optical Characteristics

**Fig. 6:**
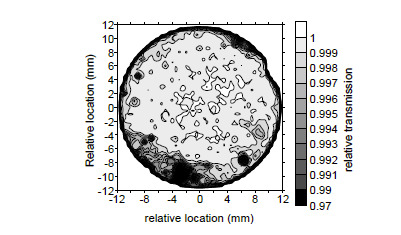
Spatial transmissivity of the legacy C4-1 window normalized to the transmissivity at the geometric center of the window.

The C-calorimeter windows are uncoated UV fused silica (SiO_2_), wedged at a 1° angle to preclude interference effects from effecting transmission. The incident laser beam is near-normal to the window and therefore insensitive to polarization. Mechanical defects such as voids and polishing artifacts yield scatter. The spatial uniformity was evaluated by scanning a 1 mm (1/e^2^) diameter, 657 nm wavelength laser across the window. The relative transmission is the ratio of the average power measurement at the geometric center to the power at an arbitrary point. The window transmissivity illustrated in Fig. 6 suggests the internal transmissivity is uniform to better than 0.02% within the central 2/3 of the clear aperture.

Window internal transmission is dominated by SiO_2_ absorption features. Hydroxide and carbon monoxide (CO) impurities produce absorption features [17, 18] that are pronounced in the near-infrared spectrum. Typically, moderate OH^−^ absorption features are observable at 1.23 µm and 1.38 µm, with the spectrally broadened OH^−^ absorption features at 2.2 µm and 2.7 µm observable at wavelengths longer than 1.7 µm [17]. Lesser absorption features attributable to hydroxide spectral overtones are present across the optical transmission range of SiO_2_ glass [17]. Metal as well as hydroxide impurities strongly affect the refractive index near the 200 nm ultraviolet (UV) cutoff wavelength [19, 20, 21]. Owing to these many absorption mechanisms, internal transmission should be measured for a given window, particularly in the regions where the above mechanisms are present.

**Fig. 7:**
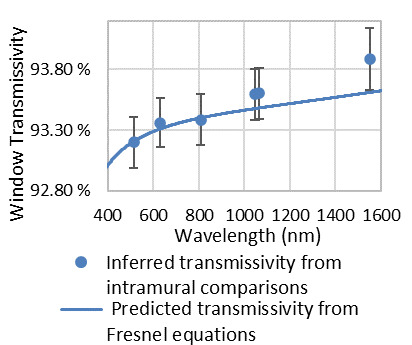
Window transmissivity inferred from comparison of the assembled calorimeter against the cryogenic radiometer transfer standards and predicted transmissivity from Fresnel reflection. Error bars are *k* = 2 expanded uncertainty.

Well separated from the strongest absorption features described above, Fresnel reflection at the air-glass-vacuum interfaces dominates window transmissivity in the ~300 nm to ~1300 nm region. As Ref. [22] reported, agreement in refractive index between various samples to better than 500 ppm Fresnel reflectance provides a reliable prediction of transmission over the region in which it is dominant. However, outside of this region, it cannot not be employed to determine window transmissivity at the level of precision needed for the C-calorimeter nor other primary standard radiometers, owing to melt-to-melt variation in impurities. Sellmeier coefficients for “typical” fused silica [22] are used to predict window transmissivity in conjunction with the Fresnel equation and treatment of higher-order internal reflections within the window. Prior validating measurements of C4 instrument window transmissivity were reported in Ref. [23]. These measurements served only for validation. Predicted transmission is shown alongside transmission inferred from new measurements in Fig. 7.

**Fig. 8:**
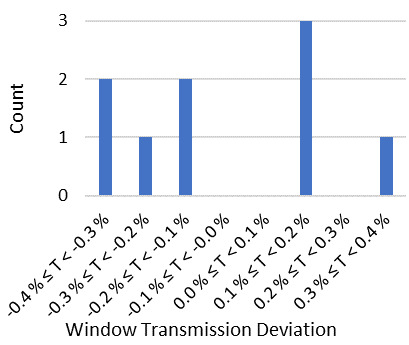
Histogram of transmission deviation inferred by comparison of the assembled calorimeter against the cryogenic radiometer transfer standards.

Surface scatter precluded direct measurement of absolute window transmissivity. Therefore, we adopted the Fresnel equations in conjunction with well-established Sellmeier coefficients from Ref. [22] to predict window transmissivity. The results of this prediction compared favorably with the window transmission inferred from comparison of the assembled calorimeter against cryogenic radiometer transfer standards. Results are shown in Fig. 7. A histogram of the deviation between prediction and inference is shown in Fig. 8. The comparison against cryogenic radiometer transfer standards was used only to evaluate uncertainty.

### 5.3 Electro-Optical Inequivalence Term

Electro-optical inequivalence is the difference between the corrected thermal rise produced by optical (laser) heating and that produced by electrical heating. We defined the electro-optical inequivalence as

**Table:** 

$I_{ineq}=100\%\times \left(1-\frac{{∆T}_{opt}}{{∆T}_{elec}}\right)$	(3)

where we compared the corrected temperature rises ${∆T}_{opt}$ and ${∆T}_{elec}$ obtained by optical and electrical heating, respectively, with the same power/energy. Unlike cavity absorptivity and window transmissivity, this term cannot be directly measured, so it must be inferred or modeled.

A previous effort attempted to infer the electro-optical inequivalence through differences in the propagation delay of optical heating versus electrical heating [8]. Though identically constructed, according to the model described in Ref. [8], the inequivalence for C4-1 was established at 0.15% (more sensitive to electrical heating than optical heating), while that for C4-4 was negligible. The numerical method in Ref. [8] used to infer the electro-optical inequivalence from the two-time-constant model was applied to the present data. This technique proved overly sensitive to the choice of rating (time) periods. As such, the results were not considered sufficiently reliable to present here.

Another previous effort used a conventional thermal analysis based on the finite element method (FEM) and established the inequivalence at less than 0.05% for both calorimeters [24]. This previous FEM analysis of heat flow within the calorimeter cavity [9] suffered from limited mesh size and simplistic boundary conditions, owing to the tools available at the time. As such, a new FEM model was constructed, taking advantage of current tools.

**Fig. 9:**
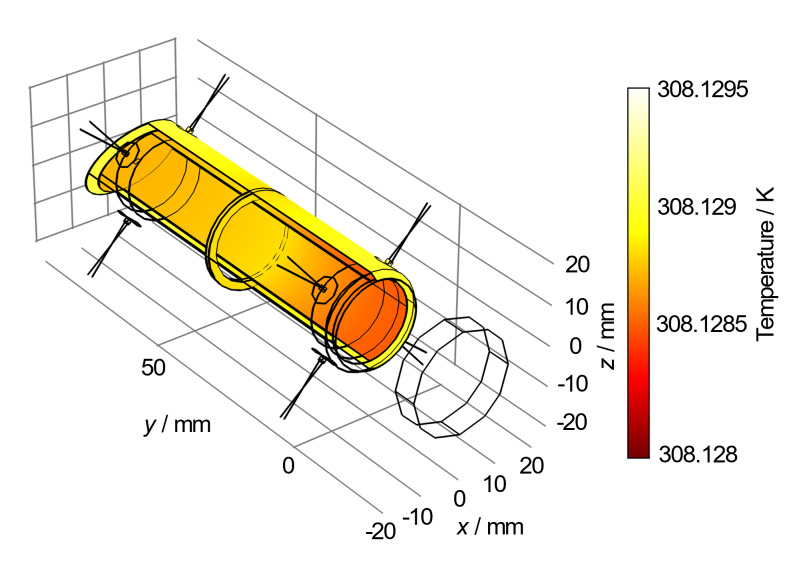

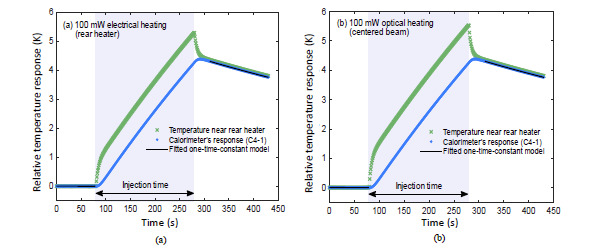
C-calorimeter’s steady-state heat map when no electrical or laser power is applied. Thermopiles are depicted by radial lines. The placement of this cavity and window within the instrument vacuum enclosure is depicted in Fig. 1. The unshaded element 20 mm in front of the cavity entrance depicts the fused silica wedged vacuum window.

The new model presented in Fig. 9 describes the cavity as two concentric cylinders with the back walls inclined by 30° from normal. The outer length of the cavity along the centerline is 90 mm, the inner cylinder is 20 mm in diameter, and the outer cylinder is 24 mm in diameter. The only mechanical contact connecting the cylinders is a ring-shaped joint at the halfway point of the cavity. The cylinders and the joint are electroformed silver, and all surfaces are gold-coated, except for the innermost surface, which is painted with diffuse black paint, forming a light-absorbing cavity. In the 1996 version of the model, the cavity cylinders were modeled as two-dimensional (2D) shells. In our model, we used cylinders with solid walls, which yields superior accuracy with a now affordable increase in computational load. Our model also accounts for thermal conductance of solids, and radiative heat transfer between surfaces and between surfaces and the isoperibol environment.

In the (physical) C-calorimeter, temperature rise is measured as a voltage with a 16 junction, type E thermopile, the reference junctions of which are connected to a temperature-controlled case stabilized to 35 °C, and the measuring junctions of which are connected to both ends of the outermost cylinder at equal spacing (illustrated in Fig. 9). With the model, the temperature was observed directly (in thermal units) at the corresponding locations on the outermost cylinder.

The correction factor *K* for laser energy measurements is obtained by electrical power/energy substitution

**Table:** 

$K=\frac{P_{\text{elec}}∙∆t}{\Delta V_{T}}$	(4)

where $P_{elec}$ is the known electrical heating power, $∆t=t_{x}-t_{2}$, and $∆V_{T,elec}={∆T}_{elec}∙b$. The thermopile’s sensitivity b is approximately 0.992 mV/K. Since the calibration factor *K* of the calorimeter describes the internal energy, it can be estimated from the heat capacity of the cavity

**Table:** 

$K=\frac{m∙c_{p}}{b}$	(5)

when the mass *m* of the cavity and the specific heat $c_{p}$ of the material are known. The functionality of the model was validated by comparing the predicted sensitivity *K* = 4.324 J/mV and time constant τ = 915 s obtained from the simulations to the experimental values of 4.236 J/mV and 785 s for electrical heating by 100 mW for 200 s. The present model differs modestly from the physical object because the precise cavity geometry differs, which affects *K*. The emissivity values of the gold-plated surfaces ($\varepsilon_{\text{Au}}$ was approximated as 0.02), affecting modeled τ, are not perfectly established. Prior simulation work in Ref. [24] established similar sensitivity. The prior and present models are adequate to the simulation task, as the difference in electro-optical inequivalence scales weakly with moderate geometry changes, while the sensitivity coefficient varies strongly [24]. As such, the modest difference in sensitivity implies negligible difference in the simulated electro-optical inequivalence versus the physical instrument.

The electro-optical inequivalence defined in Eq. (3) arises from the difference in spatial heat distributions depending on the heat source. Rear electrical heater wires are wrapped around the cavity, whereas most power of an incident laser is absorbed by the back wall of the cavity. Although the C-calorimeter’s electrical heating and optical heating occur in different locations, the high thermal conductivity of the silver cavity and thermocouple junctions located far from both heat sources ensure that the C-calorimeter’s electro-optical inequivalence is small. Figure 10 shows the simulated responses when 100 mW power is injected starting at 80 s and terminating at 280 s. Based on the differences in the simulated corrected temperature rises, the electro-optical inequivalence is approximately 0.03%. This agrees with the electro-optical inequivalence result of *less than* 0.05% modeled in 1996.

**Fig. 10:** Time-dependent response of C-calorimeter C4-1 to electrical heating (a) and optical heating (b) plotted as blue circles. The absorber near the heater (green crosses) heats and cools faster than the outermost surface of the cavity structure. The one-time-constant model in Eq. (1), presented as black lines, was fitted to the calorimeter’s response. **Table:** 

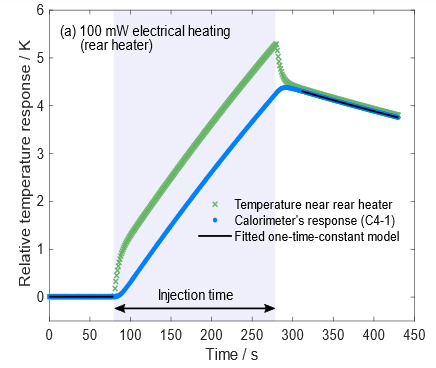 (a)	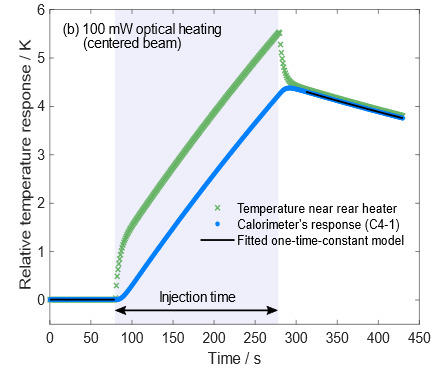 (b)

## 6 Discussion

### 6.1 Evaluating Uncertainty

The results of previous and current work establishing the instrument parameters are displayed in Table 1 for reference. With the new measurements, we are able to present the spectral absorptivity of the cavity. It shows weak spectral dependence, as illustrated in Fig. 5. By inspection, the uncertainty in spectral absorptivity is bounded by a relative rectangular distribution width, $\delta_{rel}$, of 0.01%; therefore, we ascribe uncertainty, u, of 0.0058%, where $u=\delta_{rel}/\sqrt{3}$. Electro-optical inequivalence is treated as an uncertainty. This is allowable for terms much smaller than the total uncertainty. As such, with a $\delta_{rel}$ of 0.03%, we ascribe uncertainty u of 0.017%. These results are compared to previous terms in Table 1.

Because of the vacuum grease found on the window, the prior agreement between laboratory intercomparisons deserves examination. When the analytical package currently used with the C-series instruments was under development, the LOCR [9] and ensemble of transfer standards [10] were not yet available to validate the C-series instrument performance. However, at least one transfer standard calibrated against the high-accuracy cryogenic radiometer [25] was available to the operator/programmer. The unsupported discrepancy in the cavity absorptivity from 514 nm to 1550 nm illustrated in Fig. 5 and summarized alongside prior published results in Table 1 was approximately 0.3%. The postcleaning increase in calorimeter responsivity illustrated in Fig. 4 is similar in magnitude but opposite in direction. This suggests that a previous operator used the transfer standard noted above to select a cavity absorptivity that resolved the inequivalence.

Owing to the difficulty in measuring the profile of scattered light, window transmissivity remains an irreducible source of uncertainty. The ensemble of deviation results shown in Fig. 6 is bounded by a relative triangular distribution width, $\delta_{rel}$, of 0.64%; therefore, we ascribe uncertainty, u, of 0.26%, listed in Table 1, where $u=\delta_{rel}/\sqrt{6}$. This large term motivates window replacement with improved optical glass having a polish superior to commercial-grade optics. Until the optics are upgraded, we use this uncertainty.

**Table 1:** Calibration constants and uncertainty assessed in this work.

	Value previously published	Value previously used	Value assessed in present work	$u_{rel}$	Distribution
Cavity absorptivity	0.9998 [1]	0.9964	0.9995–0.9997 (see Fig. 5)	0.0058%	Rectangular
Window transmissivity at 1064 nm	Not published	0.9348	0.9348 (see Fig. 7)	0.34%	Triangular
Electro-optical inequivalence	C4-1: 0.15% [8] 0.05% [9] C4-4: 0.01% [8] 0.05% [9]	C4-1: 0 C4-4: 0	C4-1: 0.03% C4-4: 0.03%	0.017%	Rectangular

Uncertainty contributions accepted from prior work [1, 2] are listed in Table 2. The total expanded uncertainty U (*k* = 2) is $2\sqrt{\sum {u_{rel}}^{2}}$, and for the terms listed in Tables 1 and 2, this equates to approximately 0.84%.

**Table 2:** Uncertainty contributions accepted from prior work [1, 2].

Term	$u_{rel}$* (%)*	Distribution
Electronics—stability	0.058	Rectangular
Electronics—calibration	0.018	Normal (*n* = 30)
Heater leads	0.0058	Rectangular
Inject time	0.029	Rectangular
Source stability	0.29	Rectangular
Standard meter ratio (fixed)	0.29	Rectangular
Standard meter ratio (stochastic)	Measured during each calibration	Normal (*n* varies with measurement process)
Test meter ratio		

### 6.2 Impact on Representation of the Optical Power Scale

**Fig. 11:**
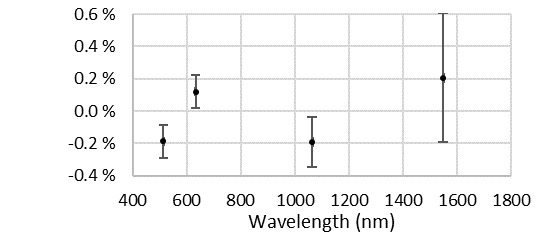
Shift in laser power scale representation provided by the C-series instruments. Positive values indicate an increase in calibration factors of customer transfer standards (where the calibration factor is the ratio of the customer instrument to the standard).

The cavity absorptivity and the electro-optical inequivalence summarized in Table 1 were then implemented for measurements provided by the C-series instruments. A comparison of system performance against the check-standards with these new values (in conjunction with optical performance improvement from window cleaning) was performed to identify the shift in scale realization. Shown in Fig. 11, the results indicate a change well within the typical 1% expanded uncertainty of the instrument system, with negligible impact upon calibration users.

### 6.3 Future Scale Representation for Optical Power

To provide scale realization at the optical power levels required for laser power meter calibrations, the use of room-temperature primary standard calorimeters at NIST appears to be unique amongst national metrology institutes (NMIs) [26]. Other NMIs favor use of cryogenic radiometers [27, 28] in conjunction with a chain of power meters capable of operation at higher (and lower) powers for scale realization. Cryogenic radiometry has several key advantages over room-temperature radiometry that enable optical power scale realization with uncertainty typically below 0.05% [9]. A cryogenic radiometer is however typically limited to operation below 1 mW, and for that reason, high power scale realization is typically achieved by electrically or optically linearized [29] thermopiles and electro-optical inequivalence measurement. Scale realization at the 1 W level with uncertainty below that achieved by the NIST C-calorimeter system has been reported by other NMIs using this scheme [26].

NIST is at present pursuing a room-temperature scale representation capable of operating across the power range observed with present calorimeter-based instruments. The new absolute room-temperature radiometer under development [30] has the potential to cut uncertainty to roughly 0.1% to 0.2% (*k* = 2). As this instrument is absolute and does not rely upon a cryogenic radiometer for calibration, we are spared the expense in labor and material required to maintain a cryogenic radiometer optimized for laser power calibrations.

## 7 Conclusions

NIST’s room-temperature calorimeters continue to provide extremely stable, sensitive, low-uncertainty optical power scale realization after 45 years of operation. Validation by comparison against transfer standards and other primary standards provides an indicator of system performance and is critical to early identification of hardware or software deficiencies. Re-evaluation of system components provides an opportunity to correct known uncertainty contributions and identify previously unrecognized contributions. Also, the advance of optical window manufacturing processes presents an opportunity to decrease system uncertainty with the installation of superpolished windows in place of the legacy windows. Successor radiometers have the advantage of such optics, which, alongside improvements in fabrication processes and planar absorbers, enable faster instruments with lower uncertainty [31].
